# A Photopolymerizable Biocompatible Hyaluronic Acid Hydrogel Promotes Early Articular Cartilage Repair in a Minipig Model In Vivo

**DOI:** 10.1002/adhm.202300931

**Published:** 2023-08-18

**Authors:** Liang Gao, Riccardo Beninatto, Tamás Oláh, Lars Goebel, Ke Tao, Rebecca Roels, Steffen Schrenker, Julianne Glomm, Jagadeesh K. Venkatesan, Gertrud Schmitt, Ebrar Sahin, Ola Dahhan, Mauro Pavan, Carlo Barbera, Alba Di Lucia, Michael D. Menger, Matthias W. Laschke, Magali Cucchiarini, Devis Galesso, Henning Madry

**Affiliations:** ^1^ Center of Experimental Orthopaedics Saarland University Kirrberger Straße 100, Building 37 D‐66421 Homburg Germany; ^2^ Fidia Farmaceutici S.p.A. Via Ponte della Fabbrica 3/A Abano Terme (PD) 35031 Italy; ^3^ Institute for Clinical and Experimental Surgery Saarland University Kirrberger Straße 100, Building 65 and 66 D‐66421 Homburg Germany

**Keywords:** cartilage defects, cartilage repairs, hyaluronic acid, hydrogels, large animal model, photo‐crosslinking

## Abstract

Articular cartilage defects represent an unsolved clinical challenge. Photopolymerizable hydrogels are attractive candidates supporting repair. This study investigates the short‐term safety and efficacy of two novel hyaluronic acid (HA)‐triethylene glycol (TEG)‐coumarin hydrogels photocrosslinked in situ in a clinically relevant large animal model. It is hypothesized that HA‐hydrogel‐augmented microfracture (MFX) is superior to MFX in enhancing early cartilage repair, and that the molar degree of substitution and concentration of HA affects repair. Chondral full‐thickness defects in the knees of adult minipigs are treated with either 1) debridement (No MFX), 2) debridement and MFX, 3) debridement, MFX, and HA hydrogel (30% molar derivatization, 30 mg mL^−1^ HA; F3) (MFX+F3), and 4) debridement, MFX, and HA hydrogel (40% molar derivatization, 20 mg mL^−1^ HA; F4) (MFX+F4). After 8 weeks postoperatively, MFX+F3 significantly improves total macroscopic and histological scores compared with all other groups without negative effects, besides significantly enhancing the individual repair parameters “defect architecture,” “repair tissue surface” (compared with No MFX, MFX), and “subchondral bone” (compared with MFX). These data indicate that photopolymerizable HA hydrogels enable a favorable metastable microenvironment promoting early chondrogenesis in vivo. This work also uncovers a mechanism for effective HA‐augmented cartilage repair by combining lower molar derivatization with higher concentrations.

## Graphical abstract

Cartilage defects are an unsolved clinical challenge. Photocrosslinkable hyaluronic acid (HA)‐based hydrogels can be applied to defects in a large animal model treated by microfracture and polymerized in situ, where they establish a favorable metastable microenvironment supporting early cartilage repair. Combining lower molar derivatization with higher concentrations is especially beneficial for such HA hydrogel‐based cartilage repair.

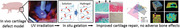

## 1 Introduction

Injuries to the hyaline articular cartilage, a composite biohydrogel containing type‐II collagen and the glycosaminoglycans chondroitin sulfate, keratan sulfate, and hyaluronic acid (HA), are a serious and challenging clinical problem. If untreated, focal articular cartilage defects may induce osteoarthritis (OA), a primary cause of chronic disability.^[^ ^1^ ^]^ An urgent clinical need exists for an advanced biomaterial that adapts to the geometry of a cartilage defect, enables rapid bonding and supports chondrogenesis while combining a high level of biocompatibility with biodegradability.^[^ ^2^ ^]^ Injectable photopolymerizable hydrogels are fascinating candidates for such regenerative approaches as they can be precisely applied to and retained in cartilage defects in situ by minimally invasive procedures.^[^ ^3^, ^4^, ^5^ ^]^

Marrow stimulation such as microfracture (MFX) of the very common symptomatic small cartilage defects is clinically efficient, although results may decline over time.^[^ ^6^ ^]^ MFX is performed arthroscopically by repeatedly perforating the previously debrided and exposed subchondral bone plate with a conical awl. A blood clot fills the defect, where mesenchymal stromal cells (MSCs) arising from the subchondral bone marrow through these bony canals undergo chondrogenesis and fibrocartilaginous repair. Enhanced techniques of marrow stimulation applying biomaterials have been proposed, aiming to improve stability of the blood clot, MSC differentiation and repair^[^ ^7^ ^]^ while also protecting the integrity of the punctuated subchondral bone during its restoration.

HA plays an important role in tissue remodeling in development, homeostasis, and disease, among which cell migration, cell–cell adhesion, and cell differentiation.^[^ ^8^ ^]^ It is a central compound of the articular cartilage extracellular matrix (ECM), lubricating the cartilage surfaces in synovial joints.^[^ ^9^ ^]^ HA‐based hydrogels provide for a 3D environment supporting chondrogenic differentiation, mimicking structural and functional properties of the ECM.^[^ ^3^, ^10^ ^]^ Photocrosslinked HA‐based hydrogels are eminent candidates because they not only imitate the environment of the cartilage ECM,^[^ ^3^, ^11^ ^]^ but can be also polymerized in situ. Because of their intrinsic fluidity and their radical‐ and catalyst‐free safety, HA hydrogels can be applied during arthroscopy,^[^ ^12^ ^]^ easily adapting to the often irregular shape of the cartilage lesions while providing an intimate bonding to the surrounding osteochondral unit. A variety of chemical modifications addresses the reduced biomechanical properties of native HA, establishing mechanically and chemically stiffer materials, like the near UV‐photocrosslinked hydrogels based on coumarin‐functionalized HA with a triethylene glycol (TEG) spacer.^[^ ^13^ ^]^ The safety of the crosslinking technology is of critical clinical importance. Due to the need for a coupling agent or a photo/radical initiator in existing crosslinking chemistries, the potential presence of free radicals may be damaging to the adjacent tissues.^[^ ^14^ ^]^ HA hydrogels overcome intrinsic limitations of the most commonly used solid type‐I collagen scaffolds such as to instruct chondrogenesis and challenging in situ fixation. However, the effect of photocrosslinkable acellular HA‐TEG‐coumarin hydrogels on chondrogenesis following MFX remains to be determined. Moreover, a possible influence of the molar degree of substitution and concentration of HA on cartilage repair is also unknown.

**Figure 1:**
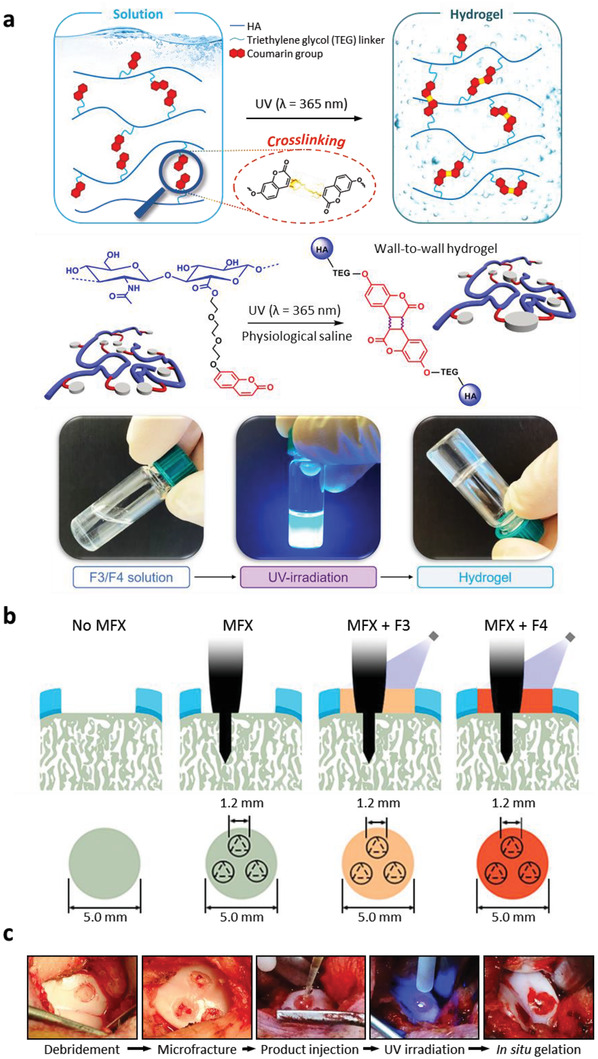
Experimental design. a) Graphical design, reaction scheme and photographic documentation of solution photocrosslinking into hydrogel. b) Full‐thickness circular chondral defects (diameter: 5.0 mm) in the trochlear groove were addressed with 1) debridement (No MFX), 2) debridement and microfracture (MFX), 3) debridement, MFX, and a hydrogel (F3) comprising 30% molar derivatization and 30 mg mL^−1^ HA (MFX+F3), and 4) debridement, MFX, and a hydrogel (F4) comprising 40% molar derivatization and 20 mg mL^−1^ HA (MFX+F4). Microfracture was standardized using a custom‐made awl (trihedral tip; diameter: 1.2 mm) to introduce three holes with a depth of 5.0 mm within the subchondral bone, evenly spaced in the circular defect. The F3 and F4 hydrogels were applied as liquids to the chondral defects and gelated in situ under UV light irradiation for 5 min at a distance of 1 cm to the basis of the defect, filling the microfracture holes too. c) Intraoperative view of the temporal sequence of the treatment. Abbreviations: HA, hyaluronic acid.

Here, we introduce a cell‐free HA hydrogel that not only promotes early cartilage repair but is also conveniently applicable as a flowable liquid and retained in situ as a gel upon photocrosslinking. We chose an uncontaminated photoinduced cycloaddition process, involving for the first time coumarin moieties to crosslink HA hydrogels within a cartilage defect in vivo. This acellular design avoids regulatory issues resulting from the clinical use of ex vivo expanded cells. We selected a clinically relevant large animal model of a full‐thickness cartilage defect treated with an established marrow stimulation technique to maximize clinical relevance. We choose an early time point of 8 weeks to provide initial guidance on whether the degradation products of the HA may initiate a pro‐inflammatory response and how these compounds perform in a situation reflecting the human condition versus appropriate controls. We investigated not only early articular cartilage repair, focusing also on the early reaction of the perforated subchondral bone during its initial phase of remodeling (**Figure** **1**). We hypothesized that HA‐TEG‐coumarin augmented MFX is superior to MFX or defects left untreated to enhance the short‐term repair of full‐thickness chondral defects in a minipig model. We also hypothesized that the molar degree of substitution and concentration of HA in this hydrogel directly affects osteochondral repair.

## 2 Results

### 2.1 Cytotoxicity and Chondrocyte Viability

**Figure 2:**
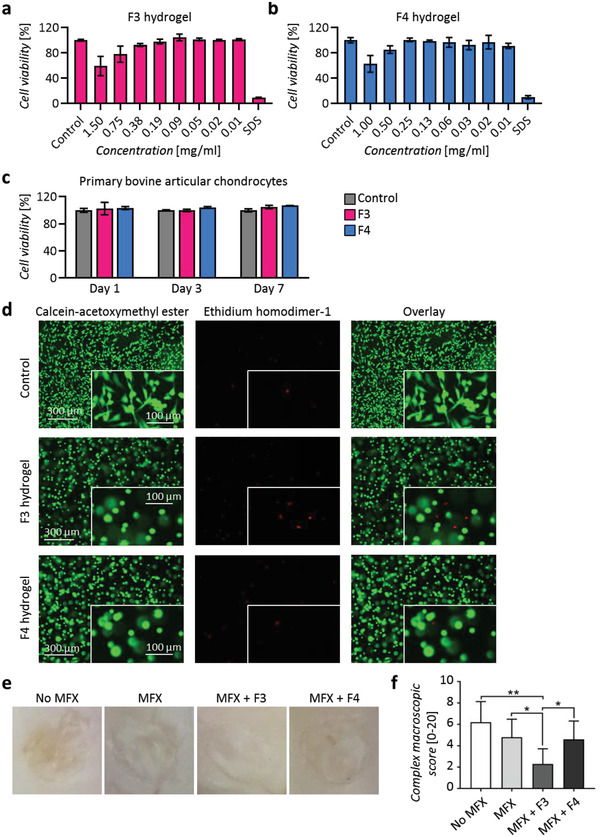
In vitro cytotoxicity, viability, and LIVE/DEAD assays, and ex vivo macroscopic analysis of cartilage defects. Alamar blue cytotoxicity assay on primary bovine articular chondrocytes after 24 h of treatment with different concentrations (mg mL^−1^) of a) F3 and b) F4 hydrogel extracts. Negative control for cytotoxicity (control): Primary bovine articular chondrocytes cultivated in DMEM/F‐12 + 10% fetal bovine serum. SDS (sodium dodecyl sulphate): positive control for cytotoxicity, primary bovine articular chondrocytes treated with 0.5 mm SDS. *n* = 4 replicates per condition. c) Primary bovine articular chondrocyte viability at 1, 3, and 7 days after encapsulation in the F3 and F4 hydrogels. *n* = 3 replicates per condition. d) LIVE/DEAD assay: Primary bovine articular chondrocytes stained with calcein‐acetoxymethyl ester (1.5 µm) and ethidium homodimer‐1 (4 µm) after 24 h of their encapsulation in control and F3 and F4 hydrogels. *n* = 3 replicates per condition. e) Representative macroscopic view of the cartilaginous repair tissue within the circular full‐thickness cartilage defects and adjacent cartilage following the four treatments in vivo at 8 weeks postoperatively. f) Complex macroscopic scores^[^ ^15^ ^]^ of the defects of the four treatment groups. *: *p* < 0.05, **: *p* < 0.01 with one‐way ANOVA and Tukey's post‐hoc test. *n* = 10 defects per group.

Cytotoxicity was assessed according to ISO 10993–5:2012 International Standard for the biological evaluation of medical devices (tests for in vitro cytotoxicity) by placing cultured primary articular chondrocytes cells directly in contact with extracts of the HA hydrogels. Similar results were obtained for both prototypes (**Figure** **2**a,b). A weak cell toxicity was registered at the first dilution tested for both hydrogels after 24 h of incubation (1.5 mg mL^−1^ for F3; 1 mg mL^−1^ for F4), whereas no cytotoxic effect was noted for all other dilutions. The viability of the articular chondrocytes encapsulated in both hydrogels was similar to that of non‐encapsulated chondrocytes at all time‐points (Figure 2c), without difference between F3 and F4. Of note, the primary articular chondrocytes preserved their viability upon irradiation and encapsulation within both hydrogels, as only a few dead cells were observed (Figure 2d).

### 2.2 Effects of Standardized In Situ Photo‐Crosslinking

Next, we assessed whether both hydrogels applied to a full‐thickness cartilage defect in vivo could be crosslinked in situ. Following microfracture, bleeding from the underlying subchondral bone was always observed and a blood clot formed within ≈3 min that entirely covered the basis of the defects. Both hydrogel solutions could intraoperatively be administered as flowable liquids, filled the focal cartilage defect and the three subchondral bone perforations induced by the microfracture procedure, and adapted to the cylindrical shape of the defect prior to the crosslinking, forming a smooth contour on its surface (Figure 1c). The hydrogels remained optically clear after defect administration and exhibited an excellent horizontal and vertical adhesion to the osteochondral tissue. After the application and constant UV light irradiation for 5 min (Figure 1c), stable in situ gelation of the hydrogels at high‐stiffness always occurred in all defects. No immediate local or systemic side effects were observed following application and irradiation, and despite the coordinated joint movements, all implants were retained in situ, without any signs of detachment or shape loss before wound closure. During necropsy, no joint effusion, macroscopic inflammation, osteophytes, adhesions or signs of hydrogel leakage were noted in any of the treated knees.

### 2.3 Microstructural Analyses of Osteochondral Repair

At 8 weeks, no remnants of the hydrogels were detectable within all defects upon qualitative macroscopic and histological examination. Semi‐quantitative macroscopic analysis of articular cartilage repair^[^ ^16^ ^]^ identified no significant differences of the total score and all individual categories between sole defect debridement (No MFX) and MFX. MFX+F3 treatment yielded significantly better macroscopic “repair tissue surface” than No MFX, significantly superior “graft level” than No MFX, MFX, and MFX+F4, and significantly better “integration with the adjacent cartilage” than No MFX and MFX. MFX+F3 therapy significantly enhanced the total cartilage repair score (6.10 ± 0.74) compared with the other 3 groups (No MFX, 1.90 ± 1.29; MFX, 2.80 ± 0.92; MFX+F4, 3.90 ± 1.52; all *p* < 0.05 compared with MFX+F3) (**Table** **1**, Figure 2e). Compared with No MFX, both MFX+F3 and MFX+F4 therapy significantly improved the total macroscopic cartilage repair score. MFX+F3 also significantly improved defect repair compared to the other 3 groups as determined by another complex macroscopic score (Figure 2f).

**Table 1:** Macroscopic grading of the repair tissue and integration.

Category	No MFX	MFX	MFX+F3	MFX+F4	*p* values
Graft level	0.20 ± 0.63	0.40 ± 0.52	1.80 ± 0.42	0.80 ± 0.92	††, §, ε
Adjacent integration	0.20 ± 0.42	0.50 ± 0.53	1.60 ± 0.52	1.10 ± 0.57	††, #, §
Surface	0.50 ± 0.71	0.80 ± 0.42	1.40 ± 0.52	0.80 ± 0.42	††
Graft color	1.00 ± 0	1.10 ± 0.32	1.30 ± 0.48	1.20 ± 0.42	n.s.
Total score	1.90 ± 1.29	2.80 ± 0.92	6.10 ± 0.74	3.90 ± 1.52	††, #, §, ε

Macroscopic evaluation of the repair tissue applying a validated elementary grading system (0 = no repair; 8 = normal articular cartilage).^[^ ^16^ ^]^ Values are expressed as the mean ± SD. *p*‐values were reported in the following manner: ^†^ *p* < 0.05 for No MFX versus MFX; ^††^ *p* < 0.05 for No MFX versus MFX+F3; ^#^ *p* < 0.05 for No MFX versus MFX+F4; ^§^ *p* < 0.05 for MFX versus MFX+F3; ^&^ *p* < 0.05 for MFX versus MFX+F4; ^ε^ *p* < 0.05 for MFX+F3 versus MFX+F4; n.s., not significant. Abbreviations: No MFX, debridement only; MFX, debridement and microfracture; MFX+F3, debridement, MFX and F3 hydrogel; MFX+F4, debridement, MFX and F4 hydrogel.

**Figure 3:**
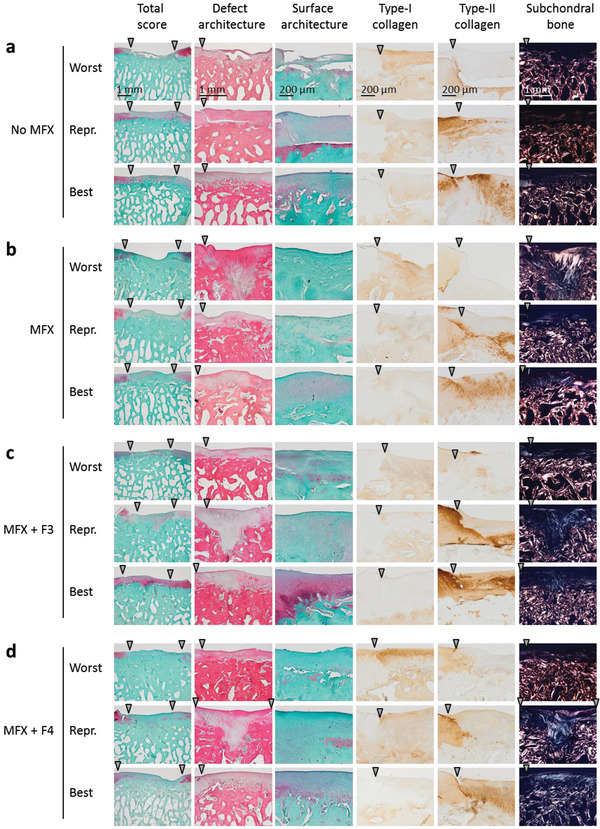
Representative histological sections. Representative histological view of cartilaginous repair tissue within the cartilage defects from the groups a) No MFX, b) MFX, c) MFX+F3, d) MFX+F4 stained with Safranin‐O/fast green or hematoxylin‐eosin, or evaluated for a positive immunoreactivity to type‐I and type‐II collagen or by polarized light microscopy at 8 weeks postoperatively. Images show the best, median (representative) and poorest results of cartilage repair based on the rating of the categories “total score,” “defect architecture,” “surface architecture,” and “subchondral bone” of the validated semi‐quantitative cartilage repair score.^[^ ^17^ ^]^ Arrowheads indicate the margin of the defect. Abbreviations: No MFX, debridement only; MFX, debridement and microfracture; MFX+F3, debridement, MFX, and F3 hydrogel; MFX+F4, debridement, MFX, and F4 hydrogel; Repr., representative.

MFX+F3 led to the highest densities of cells in the cartilaginous repair tissue having a chondrocyte morphology, not significantly different from the other 3 groups (**Table** **2**). A validated semi‐quantitative histological analysis of cartilage repair^[^ ^17^ ^]^ following MFX alone revealed no significant differences in the total score compared with sole defect debridement (No MFX, 19.98 ± 2.99; MFX, 19.99 ± 2.41) as well as all individual parameters in defects treated with MFX. MFX+F3 therapy led to a significantly improved individual parameter “defect architecture” compared with No MFX and MFX (MFX+F3, 1.48 ± 0.93; No MFX, 2.85 ± 0.99; MFX, 2.43 ± 0.91; both *p* < 0.05 compared with MFX+F3). Similarly, the parameter “repair tissue surface” was significantly improved by MFX+F3 (MFX+F3, 1.34 ± 0.40; No MFX, 2.06 ± 0.76; MFX, 2.15 ± 0.46; both *p* < 0.05 compared with MFX+F3), showing less fibrillations or irregularities. MFX+F3 significantly improved the total histological microarchitectural score compared with all other groups (MFX+F3, 15.93 ± 2.07; No MFX, 19.98 ± 2.99; MFX, 19.99 ± 2.41; MFX+F4, 18.68 ± 2.28; all *p* < 0.05 compared with MFX+F3) (Table 2, **Figure** **3**). Positive immunoreactivity to type‐I collagen was present in the repair tissue of most of the samples, without significant differences (all *p* > 0.9999) between the treatment groups (Table 2, Figure 3). Positive immunoreactivity to type‐II collagen was detected in the cartilaginous repair tissue of almost all of the samples, although its expression was mostly weaker than in the adjacent cartilage without significant differences (all *p* > 0.9999) between the treatment groups (Table 2, Figure 3).

**Table 2:** Cell quantification and semi‐quantitative histological grading of the cartilaginous repair tissue.

Category	No MFX	MFX	MFX+F3	MFX+F4	P values
Density of round cells [mm^−2^]	467 ± 363	554 ± 439	721 ± 444	651 ± 488	n.s.
Type‐I collagen immunoreactivity	3.00 ± 0.00	2.90 ± 0.32	2.80 ± 0.42	2.90 ± 0.32	n.s.
Type‐II collagen immunoreactivity	0.90 ± 0.57	1.10 ± 0.57	1.20 ± 0.42	1.20 ± 0.79	n.s.
Filling	0.95 ± 0.92	0.76 ± 0.76	0.31 ± 0.43	0.55 ± 0.48	n.s.
Integration	1.35 ± 0.48	1.24 ± 0.22	1.28 ± 0.29	1.48 ± 0.49	n.s.
Matrix staining	3.30 ± 0.72	3.29 ± 0.44	2.79 ± 0.55	3.14 ± 0.48	n.s.
Cellular morphology	4.71 ± 0.61	4.98 ± 0.08	4.54 ± 0.87	4.90 ± 0.24	n.s.
Defect architecture	2.85 ± 0.99	2.43 ± 0.91	1.48 ± 0.93	2.19 ± 1.07	††, §
Surface architecture	2.06 ± 0.76	2.15 ± 0.46	1.34 ± 0.40	1.68 ± 0.58	††, §
New subchondral bone	0.80 ± 0.33	1.15 ± 0.49	0.60 ± 0.47	0.98 ± 0.42	§
Tidemark	3.95 ± 0.16	4.00 ± 0.00	3.60 ± 0.67	3.78 ± 0.42	n.s.
Total	19.98 ± 2.99	19.99 ± 2.41	15.93 ± 2.07	18.68 ± 2.28	††, §, ε

The density of round cells with chondrocyte morphology (rounded nuclei and clear lacuna formation), were determined in the cartilaginous repair tissue. Immunoreactivity to type‐I and type‐II collagen was scored using a semi‐quantitative score (0 = no; 1 = reduced; 2 = similar; 3 = stronger immunoreactivity compared with the adjacent cartilage). Safranin O and hematoxylin and eosin‐stained histological sections were evaluated by the semi‐quantitative grading of the cartilaginous repair tissue with an inverse scoring system for cartilage repair (score 0: normal articular cartilage; score 31: absent repair tissue)^[^ ^17^ ^]^ and applying polarized light microscopy. Values are expressed as the mean ± SD. Legends for *p* values: ^#^ *p* < 0.05 for No MFX versus MFX; ^††^ *p* < 0.05 for No MFX versus MFX+F3; *p < 0.05 for No MFX versus MFX+F4; ^§^ *p* < 0.05 for MFX versus MFX+F3; ^&^ *p* < 0.05 for MFX versus MFX+F4; ^ε^ *p* < 0.05 for MFX+F3 versus MFX+F4; n.s., not significant. Abbreviations: No MFX, debridement only; MFX, debridement and microfracture; MFX+F3, debridement, MFX and F3 hydrogel; MFX+F4, debridement, MFX and F4 hydrogel. *n* = 10 samples per group.

**Figure 4:**
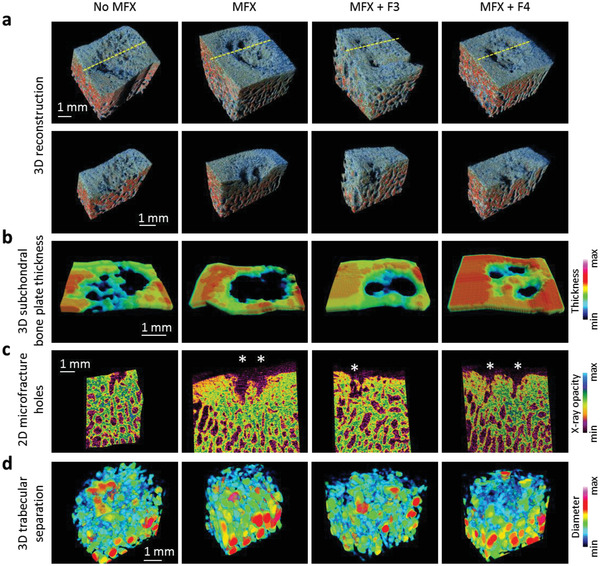
Color mapping of the subchondral bone. a) 3D reconstructed micro‐CT models of representative defects of the four treatment groups, derived from the same knee. Yellow dashed line indicates the plane where the samples were virtually cut to create the images of the second row. b) Color‐coded 3D reconstruction of the subchondral bone plate thickness. c) Color‐coded 2D micro‐CT images showing the microfracture holes (marked by asterisks). d) Color‐coded 3D reconstructed models of the trabecular thickness of the subarticular spongiosa. Abbreviations: No MFX, debridement only; MFX, debridement and microfracture; MFX+F3, debridement, MFX and F3 hydrogel; MFX+F4, debridement, MFX, and F4 hydrogel.

The subchondral bone plate below all cartilage defects was characterized by an attenuation of its microstructural parameters as assessed by micro‐CT, including BV/TV (*p* < 0.001), BS/BV (*p* = 0.007), BS/TV (*p* = 0.004), and Po(tot) (*p* < 0.001), when compared with a normal osteochondral unit irrespective of the individual treatments, without significant differences between groups. Microstructural parameters of the subarticular spongiosa were not affected (all *p* > 0.05) (**Table** **3**, **Figure** **4**). Semi‐quantitative histological analysis^[^ ^17^ ^]^ indicated that MFX+F3 hydrogel treatment significantly improved the parameter “percentage of subchondral bone” compared with MFX alone (MFX+F3, 0.60 ± 0.47; MFX only, 1.15 ± 0.49; *p* < 0.05).

**Table 3:** Micro‐computed tomography analyses of the subchondral bone plate microstructure underneath the cartilage defects.

Parameter	Treatment groups				Normal osteochondral unit	General *p* values	Specific *p* values
	No MFX	MFX	MFX + F3	MFX + F4			
Subchondral bone plate							
BV/TV [%]	18.68±11.20	13.60 ± 2.57	17.70 ± 9.74	21.32 ± 12.20	57.92 ± 5.60	<0.001	Φ, λ, γ, κ
BS/BV [1 mm^−1^]	70.82±12.65	81.36 ± 23.15	74.26 ± 16.45	66.24 ± 22.76	38.26 ± 6.43	0.007	λ, γ
BS/TV [1 mm^−1^]	12.39±5.80	10.77 ± 2.44	12.42 ± 5.90	12.38 ± 4.06	22.12 ± 3.88	0.004	Φ, λ, γ, κ
Po (tot) [%]	81.32±11.20	86.40 ± 2.57	82.3 ± 9.74	78.68 ± 12.20	42.08 ± 5.60	<0.001	Φ, λ, γ, κ
Subarticular spongiosa							
BV/TV [%]	38.29 ± 7.63	39.17 ± 8.42	33.80 ± 7.52	38. 85 ± 9.72	38.50 ± 10.99	0.394	n.s.
BS/BV [mm^−1^]	36.59 ± 10.90	36.70 ± 7.99	39.02 ± 7.51	32.99 ± 7.11	31.93 ± 9.11	0.536	n.s.
BS/TV [mm^−1^]	13.37 ± 2.13	13.83 ± 1.01	12.87 ± 2.28	12.34 ± 2.14	11.67 ± 2.24	0.345	n.s.
Tb.Pf [mm^−1^]	−18.05 ± 2.86	−20.88 ± 7.65	−14.96 ± 5.52	−16.57 ± 8.58	−13.69 ± 7.59	0.342	n.s.
SMI [‐/‐]	−0.58 ± 1.21	−0.69 ± 1.15	−0.04 ± 0.79	−0.65 ± 1.15	−0.59 ± 1.10	0.790	n.s.
Tb.Th [mm]	0.10 ± 0.3	0.10 ± 0.01	0.09 ± 0.01	0.11 ± 0.01	0.11 ± 0.02	0.467	n.s.
Tb.N [mm^−1^]	3.78 ± 0.40	3.99 ± 0.41	3.59 ± 0.68	3.58 ± 0.73	3.44 ± 0.88	0.529	n.s.
Tb.Sp [mm]	0.25 ± 0.05	0.25 ± 0.03	0.27 ± 0.05	0.26 ± 0.05	0.26 ± 0.07	0.899	n.s.
FD [‐/‐]	2.58 ± 0.13	2.54 ± 0.09	2.51 ± 0.08	2.54 ± 0.09	2.59 ± 0.07	0.555	n.s.
Po (tot) [%]	61.71 ± 7.63	60.83 ± 8.42	66.20 ± 7.52	61.15 ± 9.72	61.50 ± 10.99	0.772	n.s.
DA [‐/‐]	1.35 ± 0.16	1.30 ± 0.08	1.33 ± 0.11	1.30 ± 0.14	1.35 ± 0.10	0.879	n.s.

Micro‐computed tomography analyses of the repair of the subchondral bone plate and the subarticular spongiosa within the defect and comparison with a normal osteochondral unit. The repair tissue was sectioned into standardized volumes of interest, comprising the subchondral bone plate and the subarticular spongiosa and sized according to the surgical protocol. Values are expressed as the mean ± SD. General *p*‐value for the general comparison of each group with normal osteochondral units. Specific *p*‐values were reported as follows: ^†^ *p* < 0.05 for No MFX versus MFX; ^††^ *p* < 0.05 for No MFX versus MFX+F3; ^#^ *p* < 0.05 for No MFX versus MFX+F4; ^§^ *p* < 0.05 for MFX versus MFX+F3; ^&^ *p* < 0.05 for MFX versus MFX+F4; ^ε^ *p* < 0.05 for MFX+F3 versus MFX+F4; ^Φ^ *p* < 0.05 for No MFX versus normal osteochondral unit; ^λ^ *p* < 0.05 for MFX versus normal osteochondral unit; ^γ^ *p* < 0.05 for MFX+F3 versus normal osteochondral unit; ^κ^ *p* < 0.05 for MFX+F4 versus normal osteochondral unit. n.s., not significant. Abbreviations: BS/BV, bone surface‐to‐volume ratio; BS/TV bone surface density; BV/TV, percent bone volume; DA, degree of anisotropy; FD, fractal dimension; Po (tot), total porosity; SMI, structure model index; Tb.N, trabecular number; Tb.Pf, trabecular pattern factor; Tb.Sp, trabecular separation; Tb.Th, trabecular thickness, No MFX, debridement only; MFX, debridement and microfracture; MFX+F3, debridement, MFX and F3 hydrogel; MFX+F4, debridement, MFX and F4 hydrogel. *n* = 6–8 samples per group.

### 2.4 Principal Components Analysis and Non‐Parametric One‐Way Analysis of Similarities

**Figure 5:**
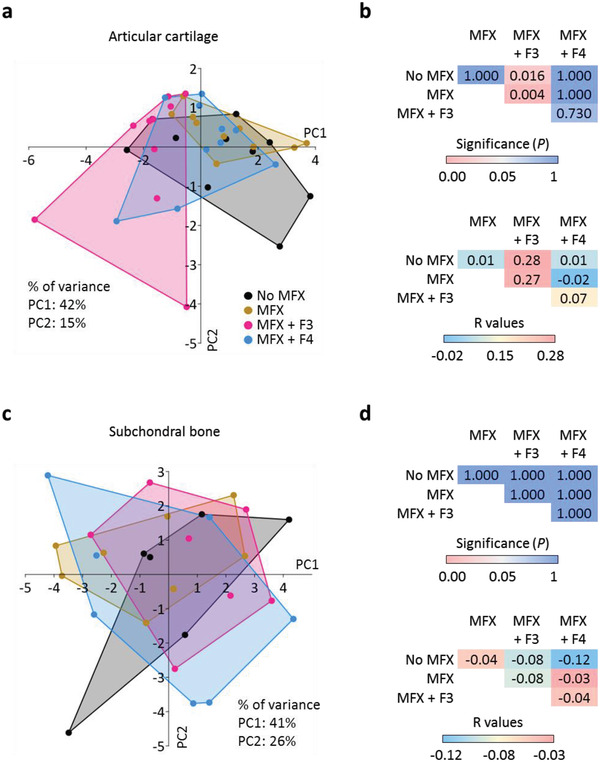
Multivariate analyses. Multivariate comparison of the a,b) articular cartilage and c,d) subchondral bone of the four treatment groups. a,c) principal components analysis (PCA) and b,d) one‐way analysis of similarities (ANOSIM) were used to compare the separation of the groups. Higher *R* values indicate larger difference between the groups, while negative *R* values indicate higher within‐group than between‐group differences. Abbreviations: No MFX, debridement only; MFX, debridement and microfracture; MFX+F3, debridement, MFX and F3 hydrogel; MFX+F4, debridement, MFX and F4 hydrogel; PC, principal component. *n* = 10 or *n* = 6–8 samples per group.

Principal components analysis (PCA) and non‐parametric one‐way analysis of similarities (ANOSIM) revealed that MFX+F3 was significantly different from No MFX (*p* = 0.016) and MFX alone (*p* = 0.004), but not from MFX+F4 (*p* = 0.730) considering all histological articular cartilage parameters (**Figure** **5**a,b). Furthermore, MFX+F4 was considerably more similar to No MFX (*p* = 1.000, *r* = 0.01) and MFX alone (*p* = 1.000, *r* = −0.02) than to MFX+F3 (*p* = 0.730, *r* = 0.07) (Figure 5a,b). None of the groups was significantly different from each other in the subchondral bone microstructure (all *p* = 1.000, *r* ≤ −0.03) (Figure 5c,d).

## 3 Discussion

This work in a clinically relevant large animal model makes several major contributions to the emerging field of injectable, in situ polymerizing regenerative therapeutics. First, in vivo application of novel photocrosslinkable HA hydrogels to articular cartilage defects is safe at short‐term follow‐up without signs of macroscopic inflammation. Second, both F3 and F4 hydrogels did not induce secondary microstructural disturbances of the subchondral bone below the treated defects compared to marrow stimulation alone. Third, and most importantly, lower molar derivatization and higher HA concentration (F3) hydrogel‐enhanced microfracture therapy significantly improved the total macroscopic and histological score compared with all other groups, besides significantly enhancing the individual repair parameters “defect architecture” and “repair tissue surface” (compared with No MFX and MFX), and “percentage of subchondral bone” (compared with MFX). The novelty of the work lies in evaluating the effects of two photocrosslinkable acellular HA‐TEG‐coumarin hydrogels on early chondrogenesis following microfracture in a large animal model. Identifying a possible influence of the molar degree of substitution and concentration of HA on cartilage repair represents the major originality.

Currently, there is a clinical need to devise regenerative strategies for cartilage repair that do not require exogenous cell manipulation. From a clinical perspective, ease of application and a short crosslinking time in situ are essential. In this context, hydrogels represent an important alternative to solid biomaterials because they are tunable in contour, allowing for a precise and minimally invasive implantation as a low‐viscosity aqueous solution into the deeply enclosed intraarticular (i.a.) location of a cartilage defect.^[^ ^18^ ^]^ The seamless self‐adaptation of the hydrogels to and integration with the mostly irregularly shaped, polygonal cartilage defects avoids the needs for trimming^[^ ^19^ ^]^ to match its shape to the defect and for suture or medical glue fixation as in the case of solid scaffolds.^[^ ^20^ ^]^ In situ, the HA solution crosslinks upon UV irradiation within only 5 min, becoming a high‐stiffness hydrogel. Photopolymerization offers several advantages over classical polymerization techniques, including temporo‐spatial control, faster curing rates at physiological temperatures, and insignificant heat generation.^[^ ^21^ ^]^ In vivo and in situ photopolymerization systems with fast time of irradiation, minimal light intensity, presence of a physiological temperature, and small levels of organic solvents can be performed when tissue and cells are present, all of particular interest for cartilage repair.^[^ ^22^ ^]^ The possible arthroscopic application also reduces the duration of recovery significantly.^[^ ^23^ ^]^

Hydrogels have excellent biocompatibility and biodegradability.^[^ ^24^, ^25^ ^]^ A freeze‐dried acellular and porous compound of a benzyl ester of HA (HYAFF) has been applied in clinical practice for cartilage regeneration for about 15 years.^[^ ^26^ ^]^ An arthroscopically injectable, articular chondrocyte‐containing hydrogel on the basis of HA and albumin that polymerizes in the cartilage defect after biocompatible cross‐linking (Novocart Inject) is clinically used since nearly 10 years.^[^ ^27^ ^]^ Another medicinal product, an HA hydrogel to deliver monolayer‐expanded allogeneic human umbilical‐cord‐blood‐derived MSCs (hUCB‐MSCs) to cartilage lesions (Cartistem), has been already tested in clinical trials.^[^ ^28^ ^]^ Additionally, the high permeability of the crosslinked hydrophilic HA polymers for oxygen, nutrients, growth factors, and other water‐soluble metabolites, as demonstrated by fluorescence recovery after photobleaching (FRAP) analysis for the HA derivative, provides a favorable metastable microenvironment that facilitates the recruitment of host cells from the bone marrow and stabilizes their differentiation, thereby enhancing endogenous regeneration.^[^ ^29^, ^30^ ^]^ Hydrogels can also be tailored to exhibit mechanical, swelling, and lubricating behaviors similar to those of articular cartilage. Swelling, one of its most relevant characteristics, is depending on a variety of aspects, among which network density (that is increasing with the irradiation time), nature of the solvent, interaction parameters between the polymer and solvent. The HA‐TEG‐coumarin compound was synthesized by conjugating coumarins to 200 kDa HA carboxylic acids via ester bonds under mild conditions. The triethylene glycol spacer represents both an ideal elastic modulus and a hydrophilicity, preserving biocompatibility at the same time, all critical to hydrogel formation.^[^ ^12^ ^]^ The kinetics of the present system of coumarin cycloaddition are very efficient. Its main advantage is that the crosslinking event, a noncytotoxic process, takes place without radical initiators or catalysts. In the area of photocrosslinked biomaterials for cartilage regeneration, this feature is essential to guarantee total biocompatibility and to minimize possible collateral reactions. The specific HA hydrogel tested here serves as a structural scaffold for the entrapment and retainment of growth factors and MSCs arising from the subchondral marrow during and after microfracture into the cartilage defect.

Following microfracture, subchondral bone changes including its resorption and formation of intra‐lesional osteophytes occur both in animal models and patients.^[^ ^31^ ^]^ Previously, in young Yucatan minipigs similarly located trochlear chondral defects were either left untreated, treated with microfracture, autologous cartilage transfer, or an acellular HA (without the additional microfracture or photocrosslinking as performed here). Already at six weeks postoperatively a cartilaginous repair tissue showed positive type‐II collagen immunoreactivity, as in the present study.^[^ ^32^ ^]^ No signs of subsidence (the process of implant migration into the subchondral bone) as sometimes reported for solid scaffolds were observed,^[^ ^33^ ^]^ despite the full weight bearing of the animals, underscoring the potential of HA as a safe structural matrix for in vivo cartilage repair. Here at 8 weeks, and also at 4^[^ ^31^ ^]^ or 6^[^ ^32^ ^]^ weeks in full thickness porcine chondral defects (both untreated, or treated with MFX or MFX+HA hydrogel), bone loss (decreased BV/TV, BS/TV, increased BS/BV) was observed in the subchondral bone plate, while the subarticular spongiosa remained intact compared to normal.^[^ ^31^, ^32^ ^]^ These findings indicate that a full thickness chondral defect (including the calcified cartilage) induces a localized osteopenia in the subchondral bone plate that negatively influences the homeostasis of the osteochondral unit, but does not affect the deeper layers of the subchondral bone. Semi‐quantitative grading identified significantly more new subchondral bone formation upon MFX+F3 treatment compared with MFX alone, while microstructural analyses revealed no significant differences between the groups. These findings suggest that both HA hydrogels did not induce secondary microstructural disturbances of the subchondral bone below the treated defects compared to marrow stimulation alone, thus permitting MSC‐based cartilage repair.^[^ ^34^ ^]^

Previous swelling tests indicated that the lower molar derivatization and higher HA content (F3) hydrogel absorbed a higher quantity of liquid than F4, likely due to the increased concentration of HA, making it more hydrophilic.^[^ ^12^ ^]^ The F3 hydrogel exhibits also superior viscoelastic properties than the F4 hydrogel.^[^ ^12^ ^]^ The hydrogel Young's moduli (bulk elastic moduli), expression of its mechanical strength,^[^ ^35^ ^]^ were of 42 ± 1 kPa and 23 ± 2 kPa for F3 and F4, respectively.^[^ ^12^ ^]^ AFM detected surface elastic moduli (*E*_surf_) equal to 213 ± 5 kPa and 173 ± 9 kPa for F3 and F4, respectively.^[^ ^12^ ^]^ These values, although far from the elasticity of native cartilage,^[^ ^30^, ^36^ ^]^ are still slightly higher than any of the collagen‐based scaffolds used for clinical cartilage repair^[^ ^37^ ^]^ and therefore represent a good compromise between sufficient mechanical resistance to clutch^[^ ^38^ ^]^ and cellular viability. Moreover, their degradation and stability have been already described.^[^ ^12^ ^]^ However, the overarching rationale for applying such hydrogels was that they are easily applicable to the (often irregular) small defects surrounded by stable normal cartilage and that are treated with microfracture, where may serve as a scaffold that can be easily colonized by MSCs and that can be absorbed in a relatively short time during chondrogenesis. Indeed, the FRAP assay, indicating the permeability of hydrogels to small molecules, revealed a diffusion cut‐off for molecules having a molecular weight that is higher than 250 kDa. Approximately 50% of the molecules inside the hydrogel were immobilized. While the [2+2] cycloaddition of coumarin moieties leads to yields in organic solvents that can be graded as low/fair,^[^ ^39^ ^]^ the structure produced by the HA (that is water‐soluble) and the TEG linker, together with the presence of hydrophobic coumarin moieties, produces a peculiar molecular arrangement in aqueous solvents that allows for short reaction times and high yields. Assuming hydrophobicity as the driving force of this photochemical cycloaddition, the coumarin moieties could be moved in very close proximity to each other, a concept resulting in a growing local concentration, therefore increasing the likelihood of reaction. In sum, such tailored hydrogels might offer an enhanced protection of the defect and its surroundings while also allowing for an additional structural support for host cell recruitment compared with conventional solid matrices.^[^ ^40^ ^]^ Moreover, the presence of TEG linker, along with the biomaterial mesoporous nature,^[^ ^12^ ^]^ makes the present derivatives suitable for i.a. drug delivery,^[^ ^41^ ^]^ overcoming the lack of cell attachment sites by tethering an RGD (Arg‐Gly‐Asp) or RGDS (Arg‐Gly‐Asp‐Ser) peptide.

A critical size cartilage defect can be defined as a being of a magnitude that does not spontaneously restore the normal osteochondral unit, in contrast to a defect of small size that spontaneously regenerates.^[^ ^42^ ^]^ The chondral full‐thickness defects established in the trochlea of the present study (round shape, measuring 5.0 mm in diameter) can be considered of critical size, since even smaller size defects (measuring 4.0 mm in diameter) do not regenerate, even at long‐term.^[^ ^43^ ^]^ Only the scenario of lower molar derivatization and higher HA concentration (F3) hydrogel‐enhanced microfracture significantly enhanced the individual repair parameters “defect architecture” and “repair tissue surface” (compared with No MFX and MFX). Notably, MFX+F3 significantly improved the total macroscopic and histological score compared with defect debridement only, MFX without or with F4. Interestingly, only two in vivo studies investigated photopolymerizable hydrogels for cartilage repair so far. Pascual‐Garrido et al. applied a polyethylene glycol hydrogel incorporating chondroitin sulfate and the cell adhesion peptide arginyl‐glycyl‐aspartic acid (RGD) without or with MSCs, whereas Lin et al. used a hybrid scaffold consisting of MSCs in a methacrylated (MA) gelatin and HA‐MA, both in lapine cartilage defects.^[^ ^44^ ^]^ The finding that the F4 hydrogel did not generate significantly better cartilaginous repair may be attributed to its inferior viscoelastic properties compared to F3, caused by the highest reachable concentration of HA in solution (20 mg mL^−1^) for F4; lower than the 30 mg mL^−1^ HA in F3. High molecular weight HA, as applied here (after photocrosslinking), is essential for ECM structural support.^[^ ^8^ ^]^ COS cells (CV‐1 in origin, and carrying the SV40 genetic material; derived from monkey kidney), produce virtually no ECM. However, if transfected to overexpress a cDNA encoding for CD44, the HA binds to the CD44. Additional parts of the matrix surround the tethered HA, forming an intricate pericellular matrix.^[^ ^45^ ^]^ Consequently, numerous ECM polymers utilize tethered strands of HA to cell surfaces, facilitating the organization of intricate structures. This phenomenon is especially notable in the chondrocyte ECM, where HA plays a crucial role in binding aggrecan, other proteoglycans, and link proteins.

The degradation product of HA may initiate a pro‐inflammatory response. Although the starting molecular weight used to produce the 2 hydrogels was about 200 kDa, the in situ crosslinking in the cartilage defect into a wall‐to‐wall hydrogel considerably increases the molecular weight of the resulting covalently crosslinked polymer that is constituted by a network of many HA chains connected to each other, with an exponential increase in the effective molecular weight. Altogether, the data suggest a mechanism that combining lower molar derivatization (30% in F3; 40% in F4) with higher concentrations of HA (30 mg mL^−1^ in MFX+F3 group; 20 mg mL^−1^ in MFX+F4 group) is essential for superior HA hydrogel‐based repair.

The major limitation of the present study is the short duration of the in vivo experiments, as the selected early time point of 8 weeks postoperatively is too short to validate the mid‐ and long‐term effects of this approach, precluding any statements that would be of clinical relevance for its long‐term effects. Thus, the significant differences between the groups at 8 weeks warrant future studies with longer time points of up to 1 year that are currently underway. Such long‐term structural and functional assessments will provide valuable data about the long‐term preservation and integrity of the repaired osteochondral unit. Also, pain and biomechanical parameters were not evaluated, and a minipig model was selected for the in vivo study, while bovine articular chondrocytes were selected for the in vitro experiments. Strengths include the clinically relevant animal model and choice of defect location, size, surgical treatment, and detailed analyses. HA scaffolds are safe and biocompatible with a long clinical track record for cartilage repair, including their utilization as cell carriers.^[^ ^26^, ^27^, ^28^, ^46^ ^]^ Because of their ease of application and safe free‐radical approach, photocrosslinkable HA hydrogels may offer not only a fascinating solution to enhance the repair of cartilage defects in patients, but also for controlled release of therapeutic molecules.^[^ ^4^, ^47^ ^]^

From a clinical perspective, it is worth noting that many HA hydrogel‐based compounds are already also accepted by the US Food and Drug Administration (FDA) as viscosupplements for intraarticular application in the context of OA, besides their clinical use for focal, non‐OA cartilage defects.^[^ ^25^, ^26^, ^27^, ^28^, ^48^ ^]^ A high molecular weight HA (physical interaction) is already in a phase III clinical trial (NCT01372475), and a non‐crosslinked HA alkylamide (physical interaction) is also registered (NCT02187549).^[^ ^48^ ^]^ Due to their advantageous characteristics, such in situ photocrosslinkable HA hydrogels could therefore move toward a clinical trial for articular cartilage repair in an efficacious manner.

In sum, the in vivo application of novel cell‐free in situ photocrosslinkable HA hydrogels in a translational large animal model enables a favorable metastable microenvironment promoting early chondrogenesis that is safe without material‐related side effects. The HA hydrogels did not induce additional microstructural disturbance of the subchondral bone compared to MFX. Combining lower molar derivatization with higher concentrations of HA represents a mechanism that leads to significantly improved early hydrogel‐based cartilage repair. The principle of combining in situ hydrogel injection and photopolymerization can be expanded to a wide range of biomedical applications.

## 4 Experimental Section

### Study Design

Hydrogels with two HA‐TEG‐coumarin concentrations were prepared (p30, i.e., F3 hydrogel, at 30 mg mL^−1^ HA‐TEG‐coumarin and p40, i.e., F4 hydrogel, at 20 mg mL^−1^ HA‐TEG‐coumarin). Full‐thickness chondral defects outlined in the trochlea of minipigs were treated with 1) debridement (No MFX), 2) debridement and standardized MFX, 3) debridement, MFX and F3 hydrogel (MFX+F3), and 4) debridement, MFX and F4 hydrogel (MFX+F4) (Figure 1). In the No MFX group, the entire layer of calcified cartilage was accurately debrided, as mandatory when preparing the defect for MFX. In the other three groups, three standardized microfracture holes were additionally introduced. Defects of the MFX+F3 group and MFX+F4 group were treated as described above, adding F3 or F4 hydrogel. These hydrogels were activated for in situ polymerization through UV light irradiation. The repair of the osteochondral unit was evaluated at 8 weeks postoperatively applying previously validated macroscopic, histological, and micro‐computed tomography (micro‐CT) analyses.

### Hydrogel Formulation and Analysis

To obtain solutions able to polymerize quickly into resistant hydrogels, 200 kDa HA‐TBA (HA tetrabutylammonium salt; Fidia Farmaceutici S.p.A., Abano Terme, Italy) was modified with coumarin moieties, using a TEG spacer, as described.^[^ ^12^ ^]^ Previous studies tested a wide range of conditions for synthesis and formulation to select the more suitable pair of HA molar derivatization degree and concentration.^[^ ^12^ ^]^ Thus, two main prototypes were considered suitable for a preclinical in vivo study: p30, an HA derivative with a molar derivatization degree of 30% (respect to HA repetitive unit), and p40, a HA derivative with a molar derivatization degree of 40%. The p30 and p40 solutions (30 and 20 mg mL^−1^, respectively), which are the basis of the F3 and F4 hydrogels, respectively, were prepared following the procedure below: 1.8 g (in the case of p30) or 1.2 g (in the case of p40) of HA‐TEG‐coumarin was added to 60 mL sterile physiological saline. Next, the mixture was stirred for 1 h until complete dissolution. The solutions were sterilized via filtration through a cellulose acetate filter (0.2 µm) and stored at 2–8 °C until their utilization. Previous studies determined the optimal irradiation time to maximize the crosslinking efficiency, considering many factors such as hydrogel stiffness (as assessed by atomic force microscopy; AFM) and mechanical compression—swelling, crosslinking degree (HPLC‐MS), hydrogel permeability to nutrients and growth factors (as assessed by FRAP), and rheological parameters.^[^ ^12^ ^]^ Furthermore, to assess the system biocompatibility and non‐cytotoxicity, in vitro tests for cellular viability and metabolism have been reported.^[^ ^12^ ^]^

### Primary Bovine Chondrocyte Isolation and Culture

Primary bovine chondrocytes were isolated from bovine stifles (the trochlear groove and femoral condyles) from skeletally mature animals. Cartilage slices were manually cut and then incubated for 1 h in DMEM/F‐12 medium containing 100 U mL^−1^ penicillin/streptomycin, 2.5 µg mL^−1^ amphotericin B (all Thermo Fisher Scientific, Monza, Italy), and 0.4% w/v pronase (Sigma‐Aldrich, Milano, Italy) at 37 °C, followed by overnight digestion at 37 °C in DMEM/F‐12 containing 0.1% w/v collagenase type II (Thermo Fisher Scientific), 2% FBS v/v, amphotericin B, and penicillin/streptomycin. Undigested cartilage was removed using a 70 µm cell strainer (Falcon, Brivio, Italy) followed by a washing step in PBS (Euroclone, Pero, Italy). The isolated articular chondrocytes were cultured under standard conditions monolayer in DMEM/F‐12, supplemented with 10% fetal bovine serum (FBS), 100 U mL^−1^ penicillin/streptomycin and 50 µg mL^−1^ l‐ascorbic acid (Sigma‐Aldrich). Every 3 days the medium was replaced. Cells from passage 1 were always used.

### Cytotoxicity Assay

Biocompatibility of the tested compounds underwent quantitative evaluation in accordance with the ISO 10993–5:2012 International Standard. For the cytotoxicity assay, F3 and F4 hydrogel extracts were prepared by photopolymerizing 1 mL of F3 and F4 sterile solutions with the UV‐lamp (*λ*_em_ = 365 nm; BTC Medical Europe S.r.l.) for 5 min in polyethylene containers (Greiner Bio‐One, Frickenhausen, Germany). These hydrogels were then added to a solution consisting of 10 mL of DMEM/F‐12 with 10% FBS and incubated for 72 h at 37 °C and 150 rpm to obtain hydrogel extracts. Primary bovine chondrocytes were cultured in monolayer (1 × 10^4^ cells per well) (96‐well plates; Sarstedt, Nümbrecht, Germany). After 24 h of incubation under standard conditions, the cells were washed with PBS. Different 1:2 dilutions of F3 and F4 hydrogel extract solutions were added to each well (4 replicates tested for each condition). Following 24 h of incubation, cells were washed, and 100 µL of complete medium containing 10% Alamar Blue (Thermo Fisher Scientific) was added. Finally, after 4 h of incubation under standard culture conditions, the amount of produced resorufin was determined with a microplate reader (excitation wavelength: 530 nm, emission wavelength: 590 nm; Nanoquant Infinite M200 Pro; Tecan, Männedorf, Switzerland). Samples were tested in four replicates. Chondrocytes in DMEM/F‐12 + 10% FBS were used as control. Chondrocytes incubated with sodium dodecyl sulfate (SDS; 0.5 mm) were used as positive control.

### Alamar Blue Assay

Cell metabolic activity after encapsulation was evaluated with Alamar Blue assay (Thermo Fisher Scientific). A total of 1.0 × 10^6^ primary bovine chondrocytes were encapsulated in 1 mL of F3 and F4 sterile solutions. 300 µL of these solutions were added to three wells of a 24‐well plate (Sarstedt) and photopolymerized as previously described to obtain wall‐to‐wall hydrogels. Then, 500 µL of complete medium was added for each well and the plate was incubated for 24 h, 3 or 7 days under standard culture conditions. To determine chondrocyte viability, the hydrogels were dissolved thanks to a treatment with hyaluronidase (*rHyal_Sk*, 52.000 U mL^−1^, Fidia)^[^ ^49^ ^]^ at 37 °C for 24 h with 5% CO_2_. After hydrogel degradation, the cells were washed with PBS, and the Alamar blue assay was performed as described in the previous paragraph. Samples were tested in three replicates. Chondrocytes not encapsulated in DMEM/F‐12 + 10% FBS were used as controls. The mean value of treated chondrocytes was normalized to the viability of untreated chondrocytes (control; set to 100%).

### LIVE/DEAD Assay

The viability of hydrogel‐encapsulated chondrocytes was also investigated with a LIVE/DEAD assay (Thermo Fisher Scientific). F3 and F4 solutions and cell encapsulation were prepared according to the procedure described in the previous sections. On the day of the assay (24 h after cell encapsulation), the cells within hydrogels were washed with PBS, and 500 µL of a 1.5 µm calcein‐AM and 4 µm EthD‐I solution in DMEM/F‐12 were added. Then, the flat black 24‐well plate (Ibidi, Glasgow, UK) was incubated for 30 min in a dark environment. Images were acquired with an inverted fluorescence microscope (DMI8, Leica Microsystems, Wetzlar, Germany). Samples were tested in three replicates.

### UV Light Irradiation

For intraoperative UV light irradiation, a high efficiency quartz‐light‐emitting diode (LED) UV‐lamp (*λ*_em_ = 365 nm, 700 mA, 120 mW cm^−2^, *Ø*_probe_ = 0.4 cm; BTC Medical Europe S.r.l., Verona, Italy) emitting a conical beam was applied. The UV‐source satisfied requirements to avoid photo‐reabsorption events by coumarin dimers, such as being monochromatic or have a cut‐off for wavelengths lower than 305 nm.^[^ ^50^ ^]^

### Large Animal Model of Photocrosslinkable Cell‐Free Hydrogel‐Enhanced Microfracture

Animal experiments were in agreement with the German legislation on protection of animals and were approved by appropriate Animal Committee (27/2017) according to German guidelines. Sample size requirements were estimated based on 80% statistical power using the two‐sample Student's *t*‐test based on comparable previous studies. Eight skeletally mature, healthy female Göttingen minipigs (age between 18 and 22 months) were sedated with an intramuscular injection of 30 mg ketamine/animal (Ketanest S, Pfizer, Berlin, Germany), 2 mg xylazine/animal (Rompun, Bayer, Leverkusen, Germany), and 1 mg atropine/animal (Braun, Melsungen, Germany), and intubated after intravenous administration of 20 mL of 2% propofol (AstraZeneca, Wedel, Germany). General anesthesia was maintained by inhalation of 1.5% isoflurane (Baxter, Unterschleißheim, Germany) and intravenous administration of propofol (6–20 mg/kg BW/h)^−1^ h^−1^). Surgical exposure using a mini‐open approach without the need for patella dislocation was performed as previously described. After the arthrotomy, circular full‐thickness chondral defects (diameter: 5.0 mm) were outlined in the trochlear groove using a dermatological punch (Kai Medical, Seki City, Japan). The entire calcified cartilage layer was meticulously removed with an oval punch (Aesculap, Tuttlingen, Germany). No bleeding from the exposed subchondral bone plate was observed prior to microfracture. Three standardized holes with a uniform diameter of 1.2 mm and depth of 5.0 mm were introduced evenly spaced in the circular defect and perpendicular to the joint surface by applying a custom‐made microfracture awl with a penetration stop (Aesculap) (groups MFX, MFX+F3, MFX+F4). Following the blood clot formation upon the microfracture, the hydrogels with two HA‐TEG‐coumarin concentrations (F3 and F4 hydrogel) were next applied to the respective defects (MFX+F3, MFX+F4). The hydrogel also filled the subchondral bone perforations induced by the microfracture procedure. The sterilized quartz‐LED UV‐lamp was secured on a tripod besides the operating table. Hydrogels were in situ polymerized for 5 min by placing the source of the conical UV beam at a distance of 1 cm to the defect. Thereafter, following a close visual inspection, the primary stability of the hydrogel implant was assessed by conducting the joint through three full ranges of motion after careful removing the UV‐lamp, followed by close visual inspection. Incisions were then closed in layers. Postoperatively, the animals were allowed immediate full weight‐bearing. A fentanyl pain patch (release rate: 100 µg h^−1^) was applied for 3 days postoperatively. If needed, 4 mg kg^−1^ BW caprofen (Rimadyl, Pfizer) were admitted orally in the first 3 postoperative days. No surgery‐related or other complication occurred.

At 8 weeks, animals were sacrificed under general anesthesia. Digital photographs of the entire defect area (*n*  =  10 per group) were taken (standardized illumination conditions). Osteochondral specimens with the defects were removed, placed in 4% formalin (1 day) and stored in 70% ethanol.

### Semi‐Quantitative Macroscopic and Quantitative Micro‐Computed Tomography Analyses

Macroscopic grading of articular cartilage repair was performed on photographs using the clinical Oswestry score initially developed for the assessment of autologous chondrocyte implantation (ACI) and cartilage repair (0 = no repair; 8 = normal articular cartilage)^[^ ^16^ ^]^ based on four parameters (graft level with surrounding cartilage, integration with surrounding cartilage, appearance of surface, and color of graft), and using a complex macroscopic score (20 = no repair; 0 = normal articular cartilage), by two independent, experienced, blinded investigators.^[^ ^15^ ^]^ The term “graft level” of the original clinical Oswestry score was kept, referring here to the “HA hydrogel graft/cartilaginous repair tissue level.” Osteochondral specimens were then scanned with a micro‐CT scanner (Skyscan 1176, Bruker, Kontich, Belgium; 18 µm isotropic resolution, 90 kV tube voltage, 278 mA current, 0.5 mm aluminum/copper filter). Scanning, reconstruction, and rotation of the image sets were performed as described previously.^[^ ^51^, ^52^ ^]^ For the evaluation of the 3D microstructure of the subchondral bone, CT Analyzer software version 1.18 (Bruker) was used. In the osteochondral unit of each defect standardized volumes of interest (VOI) were defined to separate the “subchondral bone plate” and “subarticular spongiosa.”^[^ ^52^, ^53^ ^]^ VOIs had no intersections. Similar VOIs of the normal osteochondral unit were also examined in each trochlea, adjacent to the defects but not overlapping with them. The following quantitative 3D subchondral bone parameters were evaluated within each of the four volumes of interests (VOIs):^[^ ^31^, ^54^ ^]^ bone volume fraction (BV/TV), specific bone surface (BS/BV), bone surface density (BS/TV), and total porosity [Po (tot)]. Trabecular pattern factor (Tb.Pf), thickness (Tb.Th), separation (Tb.Sp), and number (Tb.N), structure model index (SMI), fractal dimension (FD), and degree of anisotropy (DA) were only determined in the subarticular spongiosa. For 3D reconstruction of the image sets and modeling of structural thickness CTVox v. 3.2.0 (Bruker micro‐CT) was used.^[^ ^52^ ^]^

### Semi‐Quantitative Histological Analysis

Analyses were performed on sections (thickness: 4 µm) stained with hematoxylin and eosin (H&E) and safranin orange/fast green (safranin O) taken from the center of each defect in paraffin‐embedded specimen as previously described.^[^ ^31^ ^]^ A total of 320 stained sections (8 sections per defect) were analyzed using a validated semi‐quantitative histological grading system to semi‐quantitatively grade osteochondral repair. The score was based on eight parameters, which were scored individually and then combined. The total score ranged from 0 points (normal articular cartilage) to 31 points (no repair tissue).^[^ ^17^ ^]^ Round cells with a chondrocyte morphology were counted in the center of the defects (H&E, 40× magnification, *n* = 1 visual field per defect). Pictures were obtained with an Olympus BX45 microscope (Olympus, Hamburg, Germany) using the CellSens software (Olympus, version 1.12).

Immunohistochemical analyses were performed on paraffin‐embedded sections using a 1/50 dilution of a monoclonal mouse anti‐human type‐II collagen IgG (Acris Antibodies, Herford, Germany), or a 1/90 dilution of a mouse anti‐type‐I collagen IgG (Abcam, Cambridge, UK), and a biotinylated secondary anti‐mouse antibody as previously described and examined (Olympus BX45).^[^ ^55^ ^]^ Immunoreactivity to type‐I and type‐II collagen was scored blinded by an experienced investigator using a semi‐quantitative score (0 = no immunoreactivity; 1 = reduced immunoreactivity; 2 = similar immunoreactivity; 3 = stronger immunoreactivity compared with the adjacent cartilage) using the adjacent cartilage as a positive internal control.^[^ ^55^ ^]^

### Statistical Analysis

One‐way ANOVA followed by Tukey's post‐hoc test was used to compare the mean total macroscopic score. Multivariate analyses were performed for all available bone or cartilage parameters of the samples as described previously.^[^ ^52^, ^53^, ^54^, ^56^ ^]^ Principal components analysis (PCA) with a correlation matrix routine, and non‐parametric one‐way analysis of similarities (ANOSIM) with Gower similarity index were used, and considering multiplicity issues, the Bonferroni‐corrected *p*‐values were reported.^[^ ^57^ ^]^ Calculations were performed using GraphPad Prism 7.03 (GraphPad Software, San Diego, CA, USA) or Past v. 4.04.^[^ ^57^ ^]^ *p*  < 0.05 was considered statistically significant. Data are expressed as mean ± standard deviation (SD) (Figure S1, Supporting Information).

## Conflict of Interest

R.B., M.P., C.B., A.D.L., and D.G. were, at the time of the study, full‐time employees at Fidia Farmaceutici S.p.A.

## Author Contributions

H.M. and D.G. designed the study; H.M. performed animal surgery; M.D.M. and M.W.L. performed anesthesia; L.Ga., R.B., T.O., L.Go., K.T., R.R., S.S., J.G., J.K.V., G.S., E.S., O.D., M.P., C.B., and A.D.L. acquired data; L.Ga., T.O., and R.R. analyzed data; T.O., L.Ga., and R.B. prepared the figures; T.O., D.P., M.C., and H.M. interpreted data; H.M., L.Ga., and M.C. wrote the initial draft. All authors contributed to editing and revising the manuscript, and have approved the submitted version of the manuscript.

## Supporting information

Supporting Information

## Data Availability

The data that support the findings of this study are available from the corresponding author upon reasonable request.
