# Supplementary material for: A Photopolymerizable Biocompatible Hyaluronic Acid Hydrogel Promotes Early Articular Cartilage Repair in a Minipig Model In Vivo
Source: Adv Healthc Mater. 2023 Aug 18;12(26):2300931. doi: 10.1002/adhm.202300931 (PMC11468502; doi:10.1002/adhm.202300931)
Supplement: Supplementary file 1 — Supporting Information [file ADHM-12-2300931-s001.pdf]

ADVANCED HEALTHCARE MATERIALS

Supporting Information

for *Adv. Healthcare Mater.*, DOI 10.1002/adhm.202300931

A Photopolymerizable Biocompatible Hyaluronic Acid Hydrogel Promotes Early Articular Cartilage Repair in a Minipig Model In Vivo

*Liang Gao, Riccardo Beninatto, Tamás Oláh, Lars Goebel, Ke Tao, Rebecca Roels, Steffen Schrenker, Julianne Glomm, Jagadeesh K. Venkatesan, Gertrud Schmitt, Ebrar Sahin, Ola Dahhan, Mauro Pavan, Carlo Barbera, Alba Di Lucia, Michael D. Menger, Matthias W. Laschke, Magali Cucchiarini, Devis Galesso and Henning Madry**

Supporting Information

A photopolymerizable biocompatible hyaluronic acid hydrogel promotes early articular cartilage repair in a minipig model in vivo

*Liang Gao, Riccardo Beninatto, Tamás Oláh, Lars Goebel, Ke Tao, Rebecca Roels, Steffen Schrenker, Julianne Glomm, Jagadeesh K. Venkatesan, Gertrud Schmitt, Ebrar Sahin, Ola Dahhan, Mauro Pavan, Carlo Barbera, Alba Di Lucia, Michael D. Menger, Matthias W. Laschke, Magali Cucchiarini, Devis Galesso, Henning Madry**

Supplementary Table 1

Semi-quantitative macroscopic analysis of articular cartilage repair: [17]

OSWESTRY SCORE		
**Category**	**Item**	**Points**
Graft level with surrounding cartilage	Level	2
	Raised	1
	Below	0
Integration with surrounding cartilage	Complete	2
	Minor disruption (< 25 % of area)	1
	Major disruption (> 25 % of area)	0
Appearance of surface	Smooth	2
	Fine fronds	1
	Severe fronds/fibrillation	0
Color of graft	Pearly, hyaline-like	2
	White	1
	Yellow bone	0

**Total points**	**8**

Supplementary Table 2

Validated semi-quantitative cartilage repair score by Sellers et al. [18]

SELLERS SCORE		
**Category**	**Item**	**Points**
Filling of the defect relative to surface of normal adjacent cartilage	111 - 125 %	1
	91 - 110 %	0
	76 - 90 %	1
	51 - 75 %	2
	26 - 50 %	3
	< 25 %	4
Integration of repair tissue with surrounding articular cartilage	Normal continuity and integration	0
	Decreased cellularity	1
	Gap or lack of continuity on one side	2
	Gap or lack of continuity on both sides	3
Matrix staining with Safranin O-Fast green	Normal	0
	Slightly reduced	1
	Moderately reduced	2
	Substantially reduced	3
	Absent	4
Cellular morphology	(a) Normal	0
	(b) mostly round, “chondrocyte”-like cells	
	> 75 % of tissue with columns in radial zone	0
	25- 75 % of tissue with columns in radial zone	1
	< 25 % of tissue with columns in radial zone	2
	(c) 50 % round “chondrocyte”-like cells	
	> 75 % of tissue with columns in radial zone	2

	25- 75 % of tissue with columns in radial zone	3
	< 25 % of tissue with columns in radial zone (disorganized)	4
	(d) mostly spindle-shape, “fibroblast”-like cells	5
Architecture within defect (not including margins)	Normal	0
	1 - 3 small voids	1
	1 - 3 large voids	2
	> 3 large voids	3
	Cleft or fibrillations	4
Architecture of surface	Normal	0
	Slight fibrillation or irregularity	1
	Moderate fibrillation or irregularity	2
	Severe fibrillation or disruption	3
Percentage of new subchondral bone	90 - 100 %	0
	75 - 89 %	1
	50 - 74 %	2
	25 - 49 %	3
	< 25 %	4
Formation of tidemark	Complete	0
	75 - 99 %	1
	50 - 74 %	2
	25 - 49 %	3
	< 25 %	4
**Total points**		**31**
